# High-throughput metabolomics for the design and validation of a diauxic shift model

**DOI:** 10.1038/s41540-023-00274-9

**Published:** 2023-04-07

**Authors:** Daniel Brunnsåker, Gabriel K. Reder, Nikul K. Soni, Otto I. Savolainen, Alexander H. Gower, Ievgeniia A. Tiukova, Ross D. King

**Affiliations:** 1 Department of Biology and Biological Engineering, Chalmers University of Technology, Göteborg, Sweden; 2 Department of Clinical Nutrition, University of Eastern Finland, Kuopio, Finland; 3 Division of Industrial Biotechnology, KTH Royal Institute of Technology, Stockholm, Sweden; 4 Department of Chemical Engineering and Biotechnology, University of Cambridge, Cambridge, UK; 5 Alan Turing Institute, London, UK

**Keywords:** Systems biology, Biotechnology

## Abstract

*Saccharomyces cerevisiae* is a very well studied organism, yet ∼20% of its proteins remain poorly characterized. Moreover, recent studies seem to indicate that the pace of functional discovery is slow. Previous work has implied that the most probable path forward is via not only automation but fully autonomous systems in which active learning is applied to guide high-throughput experimentation. Development of tools and methods for these types of systems is of paramount importance. In this study we use constrained dynamical flux balance analysis (dFBA) to select ten regulatory deletant strains that are likely to have previously unexplored connections to the diauxic shift. We then analyzed these deletant strains using untargeted metabolomics, generating profiles which were then subsequently investigated to better understand the consequences of the gene deletions in the metabolic reconfiguration of the diauxic shift. We show that metabolic profiles can be utilised to not only gaining insight into cellular transformations such as the diauxic shift, but also on regulatory roles and biological consequences of regulatory gene deletion. We also conclude that untargeted metabolomics is a useful tool for guidance in high-throughput model improvement, and is a fast, sensitive and informative approach appropriate for future large-scale functional analyses of genes. Moreover, it is well-suited for automated approaches due to relative simplicity of processing and the potential to make massively high-throughput.

## Background

Even though *Saccharomyces cerevisiae* is a very well-studied organism, ∼20% of its proteins remain poorly characterized^1^. Many of these proteins are conserved between eukaryotes, including humans, providing a significant incentive to increase the pace of discovery.

In order to accelerate this process it will be necessary to deploy massively parallel experimental and computational methods^2,3^. Previous work has implied that the most probable path forward is via fully autonomous systems in which active learning is applied to guide high-throughput experimentation^4^. The development of tools and methods for these types of systems is therefore a research priority, as different types of biological data will provide information that will not only elucidate new functions but also vastly accelerate our knowledge of functions, enabling fully autonomous functional discovery in multiple different modalities.

Intracellular metabolomics has historically been an underutilized source of biological information, with previous studies indicating the difficulty of sample processing as a bottleneck in the way of increasing experimental throughput^5^. With rapid advances in not only lab automation but also mass-spectrometry hardware and its connected software, this is likely to change^6^. Here we investigate the potential of automated cultivation procedures and untargeted intracellular metabolomics for high-throughput functional characterization, and as a source of information for active learning based experimental design during the diauxic shift, observed in the yeast *S. cerevisiae*. Thus, in *S. cerevisiae* growing on glucose in an aerated batch culture one can commonly observe a diauxic shift: during the first growth phase, yeast ferments glucose into ethanol; when the glucose has been consumed, yeast switches to an ethanol substrate using a respiratory mitochondrial metabolism. This transition requires substantial reconfiguration of the metabolic network and a similar phenomenon can be observed in cancer cells: the Warburg effect^7^. Despite extensive research, the regulation of the diauxic shift remains poorly understood^8,9^.

Using dynamical flux balance analysis (dFBA) simulations constrained by semi-autonomously developed gene regulatory models produced by previous iterations of the robot scientist, a set of ten regulatory genes were selected due to their relevance to the shift and implications of previously unknown connections in literature^4^. These were then individually and collectively investigated using their untargeted metabolic profiles with the goal of clarifying regulatory roles and biological consequences of gene deletion. This also served as an assessment of the suitability of untargeted metabolomics as a tool for guidance in high-throughput model improvements by evaluating model fidelity.

## Results

### Metabolic profiles provide information about regulator functionality

In order to identify genes with insufficient annotation in regards to the diauxic shift and to assess the validity of the model proposed by Coutant et al., the ten strains with the highest differences in post shift growth rates with and without proposed semi-automated revisions were selected for further study^4^. The selected strains are shown in Table 1, in descending order of predicted absolute growth rate differences between the models. Three of the selections are genes without any significant functional characterization, namely: YGR067C, *RTS3* and to some extent *TDA1*. Two of them have existing homologues in humans; *DLD3* and *FAA1*.

**Table 1:** Selected deletion mutants.

Standard name	Systematic name	Protein type	Contribution	Absolute growth rate difference (|*M*1_*smart*_−*M*1|) (*h*^−1^)
YGR067C	YGR067C	Unknown	Unknown	0.00251
TDA1	YMR291W	Protein kinase	Unknown	0.000975
MEK1	YOR351C	Protein kinase	Meiotic kinase	0.000449
RTS3	YGR161C	Unknown	Unknown	0.000444
RME1	YGR044C	Zinc finger	Regulator of meiosis	0.000440
FAA1	YOR317W	LCF acyl-CoA synthetase	Fatty acid activation	0.000440
PCL1	YNL289W	Cyclin	Cell cycle progression	0.000433
GAL11	YOL051W	Transcription factor	Galactose metabolism	0.000428
OCA1	YNL099C	Putative, phosphatase	Cell cycle arrest	0.000427
DLD3	YEL071W	Dehydrogenase	D-Lactate dehydrogenase	0.000427

Details and simulated growth rate differences of deletion mutants using model paradigm and model structures proposed by Coutant et al. In descending order of absolute growth rate difference.

**Fig. 1:**
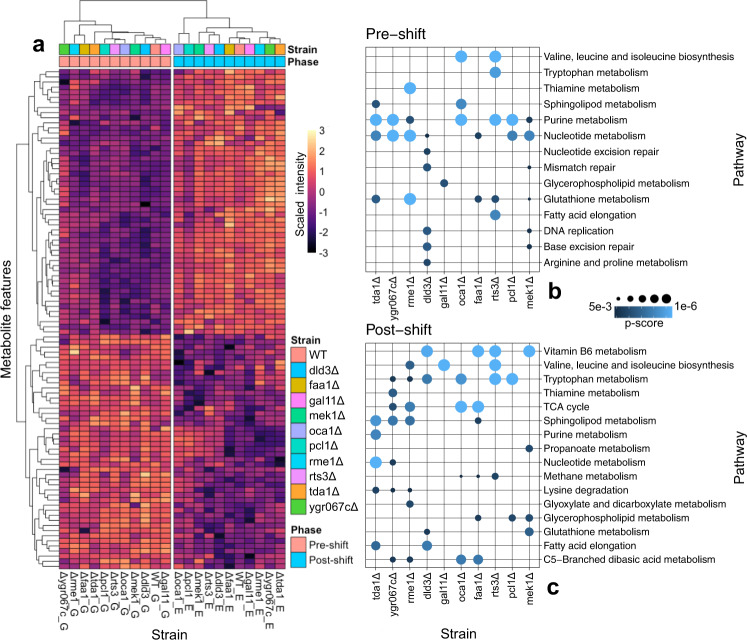
Metabolic profiles of regulatory deletants. **a** Heatmap based on normalized peak intensities for all the strains involved in the study. Selection of presented metabolites were done by selecting for features most responsible for phase-variation using discriminatory analaysis (oPLS-DA) that were deemed significant by the linear model (p-value < 0.05). Hierarchical clustering of strains was done via Pearson correlation and Euclidean distance for the metabolites. **b** Strain-wise pathway enrichment when compared to WT using FELLA (pre-shift, FELLA p-score < 0.01). **c** Strain-wise pathway enrichment when compared to WT using FELLA (post-shift, FELLA p-score < 0.01).

To identify phase-specific regulatory consequences of gene deletions, samples were taken during fermentation on glucose as well as during respiratory metabolism on an ethanol substrate for all strains in the study. An overview can be seen in Fig. 1a. The analysis makes use of pairwise comparisons with the reference strain in the same phase, and metabolite enrichment methods to contextualize the deviations^10^. The metabolic profiles of the deletion mutants showed that Pearson correlation with the reference strain was phase-dependent for the majority of the strains, indicating context-specific regulation. The correlation analysis shows the relative insignificance of the *GAL11* gene in the context of the diauxic shift and its small impact on overall metabolism in both phases. Conversely, the relative impacts of the rest of the available gene deletions are much more significant, especially in the post-diauxic growth phase.

There are also some significant differences in affected pathways between the phases, again indicating some phase-specific consequences, such as the impact on vitamin B6 metabolism post shift.

YGR067C and *TDA1* seem to have large effects on metabolism as shown by their low correlations with the reference strain. FELLA-based topological enrichment (Fig. 1b, c) shows an effect on nucleotide and sphingolipid metabolism in both phases for *TDA1*. The metabolomics data further supports the previously mentioned hypothesis, showing significant changes in abundance of sphingolipid affiliated molecules such as sphinganine and phytosphingosine. Overall, the effect of its deletion seems lower before the shift.

While less is known about YGR067C, the mode of action seems similar in the ethanol phase, with the two strains correlating highly, sharing enrichment on sphingolipid metabolism, nucleotide metabolism and lysine related processes. Although, deletion of YGR067C seems to indicate some additional involvement in central carbon metabolism, showing significant perturbations in both the citric acid cycle and C5-branched dibasic acid metabolism. Pre shift, the extent of the agitation is much lower, with most of the measured activity being in close proximity to nucleotide and purine metabolism.

Another largely uncharacterized gene, *RTS3*, is here implicated in perturbations in processes involved with amino acids such as valine, leucine and isoleucine synthesis along with tryptophan metabolism in both of the studied growth-phases. The aforementioned agitation together with the distance to the reference strain signifies a large metabolic impact, implicating further involvement.

The deletion of *MEK1* causes an agitation of several key processes connected with stress responses in adverse conditions or overall cellular maintenance. Metabolite accumulations derived from *FAA1*-deletion imply involvement in cell-membrane maintenance and the TCA cycle in the glucose phase, although overall impact seems mild after the diauxic shift, indicated by a high correlation with the reference strain. The deletant strains of *oca1*Δ and *pcl1*Δ share a degree of similarity in both of the sampled growth-phases due to a high correlation and shared biological impact on purines and tryptophan related pathways, with *OCA1* having a larger implication in more central metabolic processes.

The perturbation caused by *RME1*-deletion is significant, leading to a distinct deviation in not only the TCA-cycle and its close variations but also sections involved in valine and isoleucine synthesis in its post diauxic phase. Pre shift, the significant deviations from the reference are seemingly centered around stress-responsive elements such as glutathione metabolism and general nucleotide related metabolism.

The role of the Dld3 protein seems ambiguous, with hits in several different parts of metabolism, while still maintaining a very close connection with the reference strain in regards to the complete metabolic profile.

### Metabolic rewiring during the diauxic shift

In order to assess the metabolic impact of the diauxic shift, the entirety of the metabolomic profiles from the samples present in this study were used to recognize phase-specific changes and intricacies in the metabolic reconfiguration. Used methodologies include discriminatory analysis using orthogonal partial least squares discriminatory analysis (oPLS-DA) and third generation metabolite enrichment making use of a linear model for significance testing^10–12^.

**Fig. 2:**
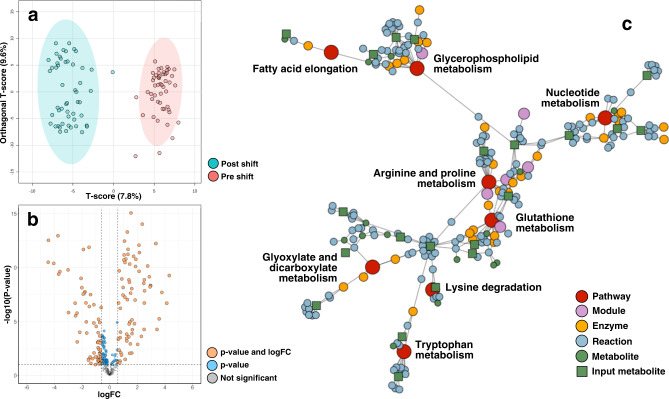
Overview of metabolomic changes across the diauxic shift. **a** Diauxic shift phase classification and 95% confidence intervals using orthogonal partial least squares discriminatory analysis (oPLS-DA) with identified peaks as features. **b** Volcano plot showing differentially expressed metabolites across the shift (raw *p*-value < 0.1 and fold-change > 1.5, linear model). **c** Diffusion based topological enrichment using FELLA with significantly enriched pathways in red (FELLA *p*-score < 0.05).

The diauxic shift comprises of a series of large cellular transformations resulting in very distinct metabolic profiles before and after the shift using the 431 distinct metabolite features present after processing. Figure 2a signifies the clear metabolite fingerprint of the phases, providing a clearly separable and reliable classification visualized with loading plots derived from the first and second component axis of an oPLS-DA projection. As seen in Fig. 2b the intensities of a large fraction of the measured species varied greatly across the phases, showing that the metabolic impact is significant and the metabolome distinctly deviated, with 215 metabolic features being significantly affected (raw *p*-value ≤ 0.1, linear model).

The significant metabolites showcased in Fig. 2a were remapped to their KEGG-identifier and subsequently used as a base for enrichment using the FELLA-algorithm. 24 out of 215 significantly expressed non-redundant metabolites were present in the intersection between the dataset and the KEGG-database on *S. cerevisiae*, see Supplementary Table 1. The metabolomics data showed that the change in phases from a fermentative to full mitochondrial respiratory metabolism is a significant one. Seemingly causing a large change in overall pathway flux, centered around essential processes such as central carbon metabolism and its connection to the switch to alternative carbon precursors such as ethanol or fatty acids. It also showed an impact on the metabolism of several different amino acids and processes essential for cell membrane viability and stress responses.

**Fig. 3:**
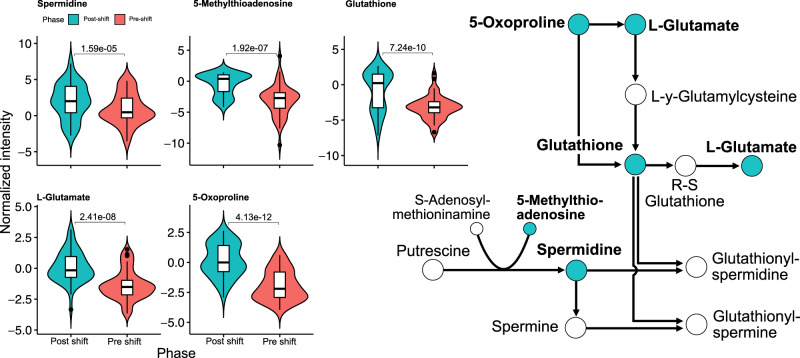
Reduced metabolic network showcasing the intersection between glutathione metabolism and arginine and proline metabolism. Highlighted metabolites are significantly accumulated over the course of the phase change as seen in the violin plots (*p*-values from the linear model). Non-highlighted metabolites were not identified in the data set. In the boxplots, the center lines represent the median; box limits represent upper and lower quartiles; whiskers extend to the first or last data point that is within 1.5× the interquartile range of the box limits in the lower and upper directions respectively. Dots are values outside of 1.5× interquartile range beyond either end of the box.

The metabolic transition resulted in the accumulation of, among others, species involved in processes implicated in stress responses. Figure 3 targets the intersection between proline metabolism and glutathione metabolism, showed as enriched in Fig. 2a. The interactions of which are sourced from KEGG^13,14^. A significant portion of the metabolites present in the intersection (L-glutamate, spermidine, glutathione, 5-methylthioadenosine and 5-oxoproline) were detected and significantly accumulated.

Overall enrichment in glycerophospholipid metabolism, as seen in Fig. 2, can be explained by significant deviation of three distinct metabolites involved in this process: choline, phosphocholine and 3-sn-glycerophosphocholine. They represent an interconnected part of metabolism, being directly associated with each other in the metabolic network.

The over-representation of tryptophan metabolism seems mainly centered around two key metabolites; kynurenic acid (4-hydroxy-2-quinolinecarboxylic acid) and indole-3-acetate, whilst tryptophan itself remains unperturbed, despite being an intermediate between the formerly mentioned metabolites.

### Autonomously improved model shows increased accuracy in predicting metabolic activity

**Fig. 4:**
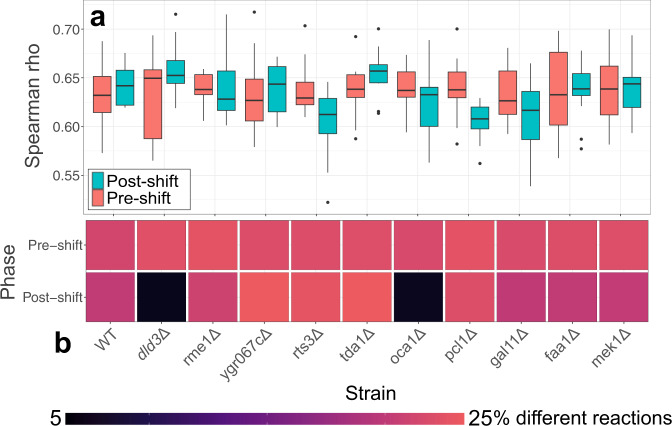
Analysis of reaction flux reroutes in M1 and M1Smart. **a** Distribution of Spearman correlations of pairwise comparisons between M1 and M1Smart simulations for knockout-strains and growth-phases. **b** Fraction of reactions significantly changed between simulations by models M1 and M1Smart (*p*-value < 0.1, Wilcoxon signed rank test). In the boxplots in **a**, the center lines represent the median; box limits represent upper and lower quartiles; whiskers extend to the first or last data point that is within 1.5× the interquartile range of the box limits in the lower and upper directions respectively. Dots are values outside of 1.5× interquartile range beyond either end of the box.

Constrained simulations according to the framework provided in Coutant et al. were analyzed to validate the revisions produced by its semi-automated model-revision approach^4^. The high amount of significantly differing reactions between the two models brought forth by the changes caused by the semi-automated method proposed in Coutant et al. in combination with the pairwise Spearman correlation (Fig. 4a) indicated that the difference in reaction flux estimates are not caused by only uniform decreases or increases of flux, but rather a reordering of reaction allocation in M1Smart when compared to the reference model, M1. Metabolite turnovers were calculated as to serve as a proxy for metabolic activity, enabling a comparison of observed species accumulation. Table 2 shows the performance of the two models when predicting for metabolite accumulation (*p*-value < 0.1 for a positive prediction and/or observation for both simulated metabolite fluxes (Wilcoxon signed-rank test) and measured species intensity (linear model)) for available metabolites in the dataset corresponding to central metabolites belonging to either central carbon metabolism or signified as an amino acid, pyrimidine, or purine.

**Table 2:** Model evaluation.

Metabolite	Model	Balanced accuracy
L-Glutamate	M1	0.265
	M1_Smart_	0.478
L-Proline	M1	0.300
	M1_Smart_	0.325
L-Histidine	M1	0.289
	M1_Smart_	0.316
L-Tryptophan	M1	0.250
	M1_Smart_	0.413
L-Leucine^a^	M1	0.000
	M1_Smart_	0.000
Adenine^b^	M1	0.000
	M1_Smart_	0.469
Guanine	M1	0.406
	M1_Smart_	0.544
Uracil^a^	M1	0.000
	M1_Smart_	0.000
Adenosine	M1	0.644
	M1_Smart_	0.544
PEP	M1	0.507
	M1_Smart_	0.382

Balanced accuracy of the different models when predicting for significant changes in metabolite flux (*p*-value < 0.1, Wilcoxon signed-rank test) in comparison to observed significant changes in metabolite accumulation (*p*-value < 0.1, linear model) for all the comparisons available in the study.

^a^no correct matches done by either of the models.

^b^no significant observations for all pairwise comparisons.).

M1Smart strictly outperformed M1 on 6 out of 10 selected metabolites, but most importantly, it predicted the activity of all the measured amino acids with higher accuracy. This seemingly showcased an improvement over the original iteration M1 using this type of evaluation. Keep in mind that neither of the models performed very well using the aforementioned metrics.

## Discussion

Some evidence indicates that functional discovery is slowing down^1^. And while some of this slowdown might be attributed to research bias, a major limitation of the methods used today is assessing functionality and generating hypotheses in a high-throughput manner while still maintaining sufficient resolution and precision. In this work, it is demonstrated that untargeted metabolomics has the potential to address this issue.

The networks of kinases and transcription factors involved in regulation represent an enormously complex system, which plays a key role in almost all cellular processes. Determination of regulator function ideally requires linking gene manipulation with global profiling of intracellular molecules (with the help of omics-based techniques). The use of targeted panels of metabolites in combination with other omics-methods and technologies may lead to subtle metabolic events being poorly investigated. By leveraging a strength of untargeted metabolomics, namely the relatively unbiased feature-collection, one can identify even small, unexpected changes in metabolism.

Untargeted metabolomics enables one to identify biochemical features of the phase-change, making it useful for classifying specific condition-based phenotypes as seen in Fig. 2a. It also enables investigation of possible modes of action and metabolic consequences of the stress connected with respiratory metabolism of ethanol. Predictably, metabolomics indirectly captured changes in glyoxylate and dicarboxylate metabolism, but also pathways heavily associated with cell survivability and fitness in adverse conditions, such as glutathione metabolism and proline metabolism. Proline and glutathione are heavily implied in the literature as biomarkers of cellular distress. The former having a role as an overall stress protectant to various forms of stressors such as heat-shock, oxidation and changes in osmolarity, while also having alternative functions as a membrane stabilizer and as an ROS (reactive oxygen species) scavenger^15^. The latter, glutathione, has roles as not only an important ROS scavenger, but also as a contributor to overall cellular fitness in adverse conditions due to, among others, its implications in cell proliferation, DNA synthesis and protein glutathionylation^16^. Some of these biological consequences and stresses are most likely subsequently shown as perturbations in nucleotide metabolism further referenced in Fig. 2a.

In the main intersection of proline and glutathione metabolism is spermidine and its reaction intermediary, 5-methylthioadenosine, both of which show significant accumulation during the respiratory phase. More specifically, spermidine is a polyamine that has previously implied relevance for membrane stabilization and enzymatic regulation, it is essential for normal cell growth, and has been implicated in human lifespan and inflammation^17,18^.

The study also further supports existing literature on the more particular effects of the shift and its connection with glycerophospholipids and local metabolic changes in activity due to changes in membrane stability^8,19,20^. Previous work has shown the importance of lipid regulation during and after the diauxic shift, especially showcasing the dynamics of glycerophospholipid metabolism^19^, something which is reinforced in this study. The change in activity is potentially caused due to stress on the cellular membrane, and an increased pressure to maintain membrane stability and relieving cell injury in an effort to keep the cells alive.

Overall, the metabolites that were found to be differentially expressed across the phases imply significant regulation of stress-responsive mechanisms, seemingly oriented mainly around ethanol exposure and accumulation of toxic metabolites produced by oxidative metabolism, such as glutathione metabolism^9,16,21^. This is also somewhat mirrored for the deletion mutants themselves, but with additional implications for several processes heavily involved in signaling, including sphingolipid metabolism^22^.

Several of the genes targeted in this study, namely YGR067C and *RTS3* are currently classified as putative proteins, whilst Tda1 is a kinase of unknown function^23^. Untargeted metabolomics provides some initial insight into their roles, indicating their involvement in essential processes such as the citric acid cycle, vitamin B6 metabolism and the metabolism of several amino acids, hopefully facilitating further investigations into their biological effect. Additionally, the Tda1 protein has, outside of this work, previously been putatively implicated in phytosphingosine metabolism, which this study strengthens further^24^.

Other genes investigated are ones with known human homologues, such as *FAA1* and *DLD3*. This study shows some implications of their deletion, such as its effect on overall stress responses, but also in pathways affecting cell-membrane stability and other processes essential for overall cellular health.

*FAA1* and *DLD3*, although being relatively well annotated genes, with main functionalities

involved in fatty acid activation and 2-hydroxyglutarate dehydrogenase, the dynamic and complex nature of regulation likely makes them involved with several additional secondary roles^25–28^. Some of which are putatively implicated here, such as the potential connection with sphingolipid metabolism, glycerophospholipid metabolism and tryptophan metabolism. There are also pathways that are affected differently when investigating strain specific differences during the different phases, such as differences in vitamin B6 metabolism only appearing post shift. This could potentially be due to its involvement in glucose and amino acid metabolism^29^.

Historically, untargeted metabolomics spectra are used as identifiable fingerprints for specific biochemical phenotypes^30^. By investigating pairwise correlation between strains, and also with the reference strain, one can infer overall similarity of the perturbation caused by the gene deletion. If we have sufficiently global enough coverage, the correlation coefficients could in this case serve as a proxy for metabolic impact, as in the case of *GAL11* which correlated highly with the reference strain in both the fermentative and respiratory phase, indicating the relative non-essentiality of the gene in the context of the diauxic shift, as seen in Fig. 1.

The use of dFBA and previously developed models and modeling frameworks in this study implies that they can be used for experiment selection and future applications in active learning and automated experimentation^4^. In this particular case, the methodology supplies informative suggestions for deletant strains, with several of the strains having previously unknown implications for the diauxic shift, and some where metabolic profile perturbations seem minimal. This discrepancy in predictions indicates the potential need for model revisions.

Model revisions made by a semi-autonomous system, as in the case of the work done in Coutant et al., where growth curves were used to infer changes in a gene regulatory network, need to be biologically validated using supplementary observations^4^. In order to assess the relevance of using untargeted metabolomics for this specific task, the metabolite accumulations were used as a proxy for metabolic activity. Comparing the model with the observations in a strictly effect-based manner shows that the revisions not only made the metabolic flux predictions more accurate, but also rerouted reaction fluxes. While other more sophisticated methods of integrating metabolomics-data into automated approaches will likely need to be developed for this purpose, this serves as a reasonable complement due to its holistic nature. The modeling paradigm of choice is essential to the form of metabolomics usage and using a FBA-centric modeling paradigm is limiting due to its steady-state assumptions regarding intracellular metabolite accumulations^31^.

In this study we investigated the potential of using untargeted metabolomics to form hypotheses regarding functionality of regulatory genes involved with the diauxic shift in the yeast *S. cerevisiae*, due to its potential use as a high-throughput form of analysis. It was also used as an indicator of model improvement for previous work done on active learning and scientific automation.

The results demonstrate that it can be used to form initial hypotheses around gene function, contextual regulation, and the phenotypical consequences of gene deletion. It was also used to characterize the diauxic shift further, showcasing supplementary modes of action for stress responses induced by fully mitochondrial respiration and ethanol exposure in yeast and giving more specific insight into the role of involved regulators. It also served as an indication of metabolic activity for use as a model validation tool. In both of these use-cases, more data in the form of different types of biological measurements would be needed to fully validate these hypotheses, but its usefulness as a high-throughput alternative to other omics-methods in order to complement computational methods in functional discovery should not be understated.

We conclude that untargeted intracellular metabolomics is well suited to generating data and hypotheses about gene function, and this could potentially be done automatically in high-throughput.

## Methods

**Fig. 5:**
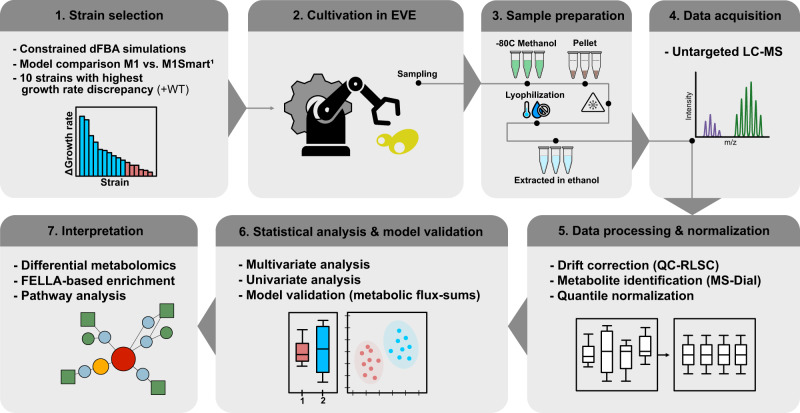
Overview on the workflow and protocols used in this study. ^1^Simulations were performed according the framework supplied in Coutant et al. and the comparison between the models served as the basis for strain selection^4^.

An overview of the workflow and methodologies used in this study can be seen in Fig. 5.

### Strains used in this study

All of the haploid yeast deletant strains present in the study were taken from the EUROSCARF deletant collection, with the strain background being *BY4741*, genotype: *MATa his3*∆1 *leu2*∆0 *met15*∆0 *ura3*∆0).

### FBA simulations and strain selection

Fully constrained dynamic flux balance simulations according the method and framework described in Coutant et al. were performed for each possible single deletion of the 682 available regulatory genes and active-learning based model combinations using identical experimental settings as in the performed experiments^4^. The difference in growth rate during the post diauxic shift phase was calculated and compared between the two models for all the deletant strains. Ten strains which were fully viable and available in the EUROSCARF collection with the largest absolute growth rate discrepancies between the revised models were then selected for further study. This to not only provide informative examples for model validation, but also to prime further investigations into their relevance to the diauxic shift.

### Growth phenotype analysis

Yeast strains from the EUROSCARF deletant collection were precultured in 250 μL YPD (yeast extract peptone dextrose) medium at 30 °C in a round bottom 96 well plate overnight. The plates were then centrifuged at 450 g for 2 min and subsequently washed in 250 μL YNB (yeast nitrogen base) before being centrifuged once more at 450 g for 2 min. Supernatant was removed and the cells were then resuspended in 250 μL of a YNB mixture, consisting of 10.5 g/L YNB without amino acids, 1.25 g/L glucose, 75 μg/L ampicillin and 0.625 g/L of L-methionine, L-leucine, L-histidine, and Uracil respectively.

A Multidrop™Combi Reagent Dispenser was used to predispense YNB in 384 well plates (Matrix, item no. 4332). A Bravo Automated Liquid Handling Platform was then used to transfer suspension from the precultures into the plate. The volume of the transfer was calculated so that an initial OD (optical density) of approximately 0.07 could be reached, using a final cultivation volume of 80 μL. The outermost quadrants of the 384 well plate were not inoculated so as to not only serve as a media control but also to avoid edge effects due to evaporation.

After plate preparation, a growth profiling protocol was created using the automated laboratory cell “Eve”^4,32^. The plate was subsequently transferred from Eve’s Cytomat™ automated incubator (30 °C) to a Teleshaker Magnetic Shaking System, where it was shaked for 20 s at 1000 rpm, divided evenly between clockwise and counter-clockwise double-orbital shaking. After shaking the plate was transferred to a BMG Polarstar plate reader, where it underwent optical density measurements at 560 nM (the temperature in the plate reader was kept at a constant 30 °C). After measuring, the plate was returned to the incubator. The protocol was automatically repeated every 20 min for up to 48 h depending on the strain.

To adjust for non-linearity, OD measurements were then background corrected and adjusted using a third degree polynomial as in Jung et al.^33^. The polynomial was derived using an OD-ladder of serial dilutions spanning the dynamic range of the OD-reader (0 − 2 OD_560_) resulting in the polynomial in Equation 1 (x = OD).

1 $$x_{Adj} = - 0.01454 + 1.231x - 0.6393x^2 + 0.4985x^3$$

The growth curves were then subsequently analyzed using AMiGA to assess the timing of the shift and the growth rates in the different phases^34^.

### Sample quenching and metabolite extraction

Strains were grown in the same conditions as in the section above. For the pre shift samples, they were collected at 0.5 OD_560_ or as close to the middle of the initial fermentative phase as possible. For the post shift samples, they were collected at 1 OD_560_ or shortly after glucose depletion when additional growth could be verified. Samples that did not adhere to this were subsequently removed after processing.

For metabolomic samples, cells were pooled and as quickly as possible transferred to 2 mL microcentrifuge tubes containing absolute methanol (99% purity) prechilled on dry ice. The ratio between sample and methanol was kept at 1:1 v/v. The tubes were kept in dry ice during the process and were as quickly as possible transferred to a centrifuge in which they were centrifuged at 2040 g for 5 min. The supernatant was then discarded, and the pellets snap frozen in −80 °C ethanol and subsequently transferred and stored at −80 °C. Samples were further freeze dried overnight and later kept at −80 °C pending further analysis.

Preparation of sQCs (study-specific quality control samples) was conducted by pooling equal amounts of each sample for the respective batches. The sQCs were subject to the same sample preparation procedure as the actual samples. sQCs were injected at the beginning, at the end, and systematically between sets of samples throughout the batch sequence.

Metabolite extraction was done by pouring 75% ethanol (preheated to 95 °C, with a ratio of 1 mL ethanol per 1 mg of sample) over the freeze-dried yeast biomass samples in a microcentrifuge tube. Samples were then immediately vortexed for 1 minute and the tube was then placed on a heater for 3 min, and allowed to cool down for 10 min at 4 °C. Further, the tubes were centrifuged for 15 min (5000 g, +4 °C) and the supernatant was transferred to another microcentrifuge tube and stored at −80 °C until analysis.

### Untargeted LC-qTOF metabolomics analysis and data processing

The analysis of samples was performed on an Agilent UHPLC-qTOF-MS system which consisted of a 1290 II Infinity series UHPLC system with a 6550 UHD iFunnel accurate mass qTOF spectrometer. During the analysis, the samples were kept at 4 °C. Analytes were separated by using reverse phase (Waters Acquity UPLC HSS T3 column (100 × 2.1 mm, 1.8 μM)) chromatography. The Agilent MassHunter workstation was used to operate and monitor the instrument and acquire data. Reversed phase mobile phase included (A) water and (B) methanol, both containing 0.04% formic acid. The linear gradient elution was: 0–6 min, 5–100% B, 6–10.5 min, 100% B. Mobile phase flow was set at 0.4 mL/min. Metabolites were ionized by a Jetstream electrospray ionization (ESI) source. The mass spectrometer was operated in positive mode. The spectrometer parameters were set as follows: drying gas (nitrogen) temperature at 175 °C and flow at 12 mL/min, sheath gas temperature at 350 °C and flow at 11 L/min, nebulizer pressure at 45 psi, capillary voltage at 3500 V, nozzle voltage at 300 V, fragmentor voltage at 175 V. Data were acquired within a 50–1600 m/z range in centroid mode with the acquisition rate set at 1.67 spectra/s. The MS abundance threshold was set at 200. Iterative MS/MS data acquisition was performed in positive mode with 10 eV, 20 eV and 40 eV collision energies and with the same chromatographic conditions as for the MS analysis. It was performed on sQC samples ensuring that spectra were collected from ions present in a significant majority of the samples. Positive mode MS2 fragmentation spectra were collection using Agilent Auto MS/MS acquisition mode with a precursor ion exclusion list of commonly occurring positive mode high-intensity precursor ions. The exclusion list is included in supplementary information (Supplementary Table 2).

Initial peak processing was done using MS-Dial (v4.7), peak detection made use of a weighted moving average method and allowed for a minimum peak-width of 5 s and minimum peak height of 2000 counts. Identification was done by manual comparison with the Riken library on both MS and MS/MS profiles when available (m/z only)^35^. Identified peaks with an average value below included blanks were removed along with peaks that had a fill beneath 0.1, signal to noise ratio below 10 and if they were not detected in two thirds in any of the experimental batches. Measured ion intensities of distinct samples taken in both phases were corrected for potential signal drifts using quality control sample based robust locally estimated scatterplot smoothing (QC-RLSC) using the NormalizeMets library (v.0.24) in R (v.4.2.1)^36^. Batches were then concatenated based on identifications, adduct type, m/z similarity (±0.01) and retention time (±0.75). Feature intensities were then normalized with quantile normalization using the preprocessCore library (v.1.52.1) using the algorithm outlined in Bolstad et al.^37^.

### Analysis of untargeted metabolomics data

Discriminatory analysis via oPLS-DA and LIMMA linear modeling was done via the MetaboAnalyst-suite (v.5.0.0)^11,12,38^. For significance testing of the identified peaks, a linear model was set up treating experimental batches as covariates, with the exception of across-shift analysis, where strain and batch was set up as covariates. The sample size for the cross-phase analysis was 102, with 52 and 50 samples in the post and pre shift phases respectively. Each strain/condition combination had between 3 and 6 replicates. See Supplementary Tables 3–22 for the full set of statistical parameters for the metabolites present in the intersection between the data set and the KEGG database derived from the linear model for each comparison. Enrichment studies were done with a diffusion based method using FELLA (v.1.14.0) where input metabolites were deemed significantly changed if below an unadjusted p-value of 0.1 derived by the linear model, similar to the approach as in Nakic et al.^10,39,40^. Identified metabolites were matched to the KEGG-database using InChIKeys supplied by MS-dial (v4.7)^13,14,35^. Correlation analysis was done using hierarchical clustering based on Pearson correlation in R (v.4.2.1), using an average across replicates.

### Model validation and identification of metabolic activity

To infer metabolic activity of specific metabolites, the basal flux-sum, Φ, was calculated for all of the metabolites present in iMM904 using Equation 2, similar to the work presented in Chung et al.^41^.

2 $${{\Phi }}_i = 0.5\mathop {\sum }\limits_j \left| {S_{ij}v_j} \right|$$

The metabolite flux-sum was calculated for all unique metabolites, *i*.

Significantly differing (unadjusted *p*-value < 0.1) simulated reactions fluxes and metabolite flux-sums were identified using a two-sided Wilcoxon signed rank test using thirteen simulations from both models as observations. To ensure that the model revisions were not only based on uniform increases or decreases of reaction fluxes, a pairwise Spearman correlation was calculated using simulations from both models.

Difference in metabolite flux-sums were then compared with measured accumulations to estimate accuracy of the flux reroutes suggested by model revisions, treating the metabolite flux-sums as predictors of species accumulation, a modification of the approach suggested in Mo et al.^42^. The metric used was balanced accuracy, to be able to manage the imbalance of activity present in the different deletion mutants and phase-changes.

### Reporting summary

Further information on research design is available in the Nature Research Reporting Summary linked to this article.

## Supplementary information

Supplementary Information

Supplementary Tables 3-22

Reporting Summary

## Data Availability

Processed data and supplementary data can be found on Github at https://github.com/DanielBrunnsaker/DShift. Raw data in the form of mzmL-files can be downloaded from Zenodo at 10.5281/zenodo.7105589.

## Code availability

All code needed to recreate the analysis and figures in the study can be found on Github at https://github.com/DanielBrunnsaker/DShift.
