# Supplementary material for: High-throughput metabolomics for the design and validation of a diauxic shift model
Source: NPJ Syst Biol Appl. 2023 Apr 7;9:11. doi: 10.1038/s41540-023-00274-9 (PMC10082077; doi:10.1038/s41540-023-00274-9)
Supplement: Supplementary file 1 — Supplementary Information [file 41540_2023_274_MOESM1_ESM.pdf]

Supplementary information

High-throughput metabolomics for the design and validation of a diauxic shift model

Daniel Brunnsåker[1,*], Gabriel K. Reder[1], Nikul K. Soni[1], Otto I. Savolainen[1,2], Alexander H. Gower[1], Ievgeniia A. Tiukova[1,3] & Ross D. King[1,4,5]

[1] *Department of Biology and Biological Engineering, Chalmers University of Technology, Göteborg, Sweden*
[2] *Department of Clinical Nutrition, University of Eastern Finland, Kuopio, Finland*
[3] *Division of Industrial Biotechnology, KTH Royal Institute of Technology, Stockholm, Sweden*
[4] *Department of Chemical Engineering and Biotechnology, University of Cambridge, Cambridge, UK*
[5] *Alan Turing Institute, London, UK*

[*] *danbru@chalmers.se*

Supplementary Tables

Supplementary Table 1. Statistical parameters for cross-phase (pre shift vs. post shift) analysis using detected metabolites intersecting with the KEGG database on S. cerevisiae.

KEGG ID	logFC	AveExpr	t	P-Value	Functional annotation in KEGG
C00253	2.1151	-0.56388	8.3132	8.4421e-13	True
C02560	1.3226	-0.32281	8.1267	2.0625e-12	False
C08370	-1.9041	0.097606	-8.1218	2.1112e-12	False
C01879	2.1481	-0.86703	7.9815	4.1264e-12	True
C03134	1.7288	-0.53257	7.8143	9.1477e-12	True
C01717	1.4448	-0.40508	7.4666	4.7457e-11	True
C07480	-3.01	-0.51481	-7.2015	1.6469e-10	True
C02320	2.8651	-2.0097	6.8831	7.2351e-10	True
C00954	0.87431	-0.059959	6.7863	1.131e-09	True
C00147	2.4584	-1.5376	6.5785	2.9288e-09	True
C14291	-2.015	-0.74106	-6.3753	7.3626e-09	False
C01046	1.5891	-0.46324	6.3288	9.0805e-09	True
C00588	2.5234	-1.0696	6.228	1.4272e-08	True
C00025	1.5123	-0.61454	6.111	2.4051e-08	True
C06343	0.96117	-0.25855	6.0233	3.5483e-08	False
C00170	2.5001	-1.7441	5.6365	1.9181e-07	True
C06343	-1.1492	-0.0090551	-5.5502	2.7766e-07	True
C00148	1.6125	-1.264	4.769	6.986e-06	True
C02727	1.4557	-1.1299	4.6472	1.1295e-05	True
C00315	1.7898	1.4669	4.559	1.5922e-05	True
C10494	-0.53427	-0.5226	-3.8978	0.00018507	False
C11874	-0.71187	0.26365	-3.8425	0.00022486	True
C00670	1.2307	-0.47112	3.7041	0.00036327	True

C00114	0.62645	-0.2936	3.6524	0.00043329	True
C01012	-0.44699	0.0020465	-3.4603	0.00082257	False
C13355	-0.84673	-0.54404	-3.4399	0.00087944	False
C07475	-0.32851	-0.26963	-3.421	0.00093517	True
C08183	-0.6734	-0.043878	-3.4014	0.00099673	False
C06414	0.42093	-0.0012884	2.5939	0.011055	False
C10120	-0.61261	0.14136	-2.5574	0.012197	False
C00371	0.19175	0.06823	2.5543	0.012298	True
C08437	-1.0232	-0.62035	-2.3713	0.019827	False
C10561	-0.67274	-0.36864	-2.3545	0.020692	False
C00163	-1.5194	-1.1467	-2.1721	0.032445	True
C00242	0.37424	-0.42955	2.1255	0.036251	True
C08626	-1.5395	-0.87497	-2.1013	0.038373	False
C18877	-0.58746	0.00043944	-2.0121	0.047157	False
C06425	-0.71554	-0.013201	-1.7887	0.076986	True
C03758	-0.18739	0.042517	-1.7045	0.091695	True
C11087	0.16784	0.18302	1.6748	0.097397	False
C06545	-0.56631	0.19685	-1.6388	0.1047	ns
C15545	0.14034	0.02898	1.6344	0.10563	ns
D00663	-0.19869	-0.056587	-1.6152	0.10973	ns
C07592	-0.42917	-0.91903	-1.6088	0.11112	ns
C14790	-0.11719	-0.011289	-1.6083	0.11122	ns
C14412	-0.1471	0.060613	-1.5067	0.13535	ns
C17762	0.45108	-5.4462	1.4803	0.14223	ns
C04294	-0.14434	-0.016097	-1.238	0.2189	ns
C00144	0.19579	0.22038	1.2277	0.22272	ns
C10702	-0.50448	-0.50521	-1.163	0.24787	ns
C08610	-0.32119	0.059887	-1.1428	0.25611	ns
C14428	-0.13227	-0.58898	-1.1401	0.25723	ns
C00106	0.10232	0.091596	1.1276	0.26246	ns
D02500	-0.15571	-0.24523	-1.1133	0.26851	ns
C08261	-0.093519	-0.053772	-1.104	0.27248	ns
C10645	0.30331	-0.20745	1.093	0.27725	ns
C10430	0.097051	-0.48387	1.0384	0.30181	ns
C01588	-0.16453	0.0041303	-1.0202	0.31032	ns
C00852	-0.248	-0.43656	-0.93486	0.35233	ns
C00495	-0.47346	-0.58054	-0.91741	0.36135	ns
C12679	0.15596	-0.064885	0.908	0.36627	ns
C03354	-0.070604	-0.059314	-0.90574	0.36746	ns
C00141	0.18939	-0.10491	0.83595	0.40537	ns
C00314	-0.085649	0.12888	-0.79866	0.42656	ns
C10331	-0.20072	-0.051982	-0.77964	0.43762	ns
C12227	0.19812	-5.2438	0.76333	0.44724	ns
C00074	0.095684	-0.096283	0.76061	0.44885	ns
C11519	-0.24608	0.032882	-0.70728	0.4812	ns
C06105	-0.081756	-0.14115	-0.64162	0.52273	ns
C00597	-0.11523	-0.21063	-0.62537	0.53329	ns
C06526	0.094473	-0.41016	0.56774	0.5716	ns
C12144	-0.089333	-0.061213	-0.53892	0.59125	ns
C14229	-0.047544	-0.14739	-0.50677	0.61354	ns
C01118	-0.11158	-0.31506	-0.4493	0.65428	ns
C02348	0.063959	0.17142	0.40938	0.68322	ns
C00417	0.035916	-0.054701	0.395	0.69376	ns
C01234	-0.028129	0.061637	-0.39112	0.69662	ns

D02500	0.082678	-0.10947	0.35644	0.72234	ns
C01996	0.054353	-0.10297	0.34246	0.7328	ns
C00768	-0.04082	0.05488	-0.27158	0.78656	ns
C00955	0.083977	-0.13342	0.22135	0.82531	ns
C06343	-0.022197	0.13313	-0.20797	0.83572	ns
C00212	0.053423	-0.86336	0.19671	0.84449	ns
C20157	-0.015491	0.05251	-0.18293	0.85526	ns
C01479	0.045039	0.47231	0.17479	0.86163	ns
C05141	0.11556	-0.80673	0.17441	0.86193	ns
C20197	-0.018317	-0.22608	-0.15437	0.87766	ns
C10472	-0.026126	-0.08686	-0.14214	0.88729	ns
C00836	-0.015821	-0.051852	-0.086497	0.93126	ns
C00123	-0.0055516	0.11414	-0.077611	0.93831	ns
C19261	-0.001685	-0.018462	-0.024834	0.98024	ns

Supplementary Table 2. Exclusion table for reference ions

On	Prec. m/z	Delta m/z (ppm)	Z	Prec. Type	Ret. Time (min)	Delta Ret. Time(min)	Iso. Width	Collision Energy
TRUE	74.06001	20	1	Exclude	0.627	13	Medium (~4 m/z)	
TRUE	85.0283	20	1	Exclude	0.969	13	Medium (~4 m/z)	
TRUE	93.05	20	1	Exclude	0.8	13	Medium (~4 m/z)	
TRUE	97.02808	20	1	Exclude	0.696	13	Medium (~4 m/z)	
TRUE	104.107	20	1	Exclude	0.666	13	Medium (~4 m/z)	
TRUE	111.05611	20	1	Exclude	0.714	13	Medium (~4 m/z)	
TRUE	113.03535	20	1	Exclude	0.948	13	Medium (~4 m/z)	
TRUE	115.05564	20	1	Exclude	0.736	13	Medium (~4 m/z)	
TRUE	116.07066	20	1	Exclude	0.733	13	Medium (~4 m/z)	
TRUE	117.02361	20	1	Exclude	1.043	13	Medium (~4 m/z)	
TRUE	119.03972	20	1	Exclude	2.686	13	Medium (~4 m/z)	
TRUE	120.06516	20	1	Exclude	0.669	13	Medium (~4 m/z)	
TRUE	124.03938	20	1	Exclude	0.765	13	Medium (~4 m/z)	
TRUE	126.99873	20	1	Exclude	0.513	13	Medium (~4 m/z)	
TRUE	130.05074	20	1	Exclude	0.67	13	Medium (~4 m/z)	
TRUE	130.06525	20	1	Exclude	1.628	13	Medium (~4 m/z)	
TRUE	132.10498	20	1	Exclude	1.469	13	Medium (~4 m/z)	
TRUE	135.02769	20	1	Exclude	1.009	13	Medium (~4 m/z)	
TRUE	136.06694	20	1	Exclude	2.685	13	Medium (~4 m/z)	
TRUE	137.0708	20	1	Exclude	2.6	13	Medium (~4 m/z)	
TRUE	146.06009	20	1	Exclude	3.888	13	Medium (~4 m/z)	
TRUE	146.16512	20	1	Exclude	0.567	13	Medium (~4 m/z)	
TRUE	147.07664	20	1	Exclude	0.777	13	Medium (~4 m/z)	
TRUE	147.07874	20	1	Exclude	0.667	13	Medium (~4 m/z)	
TRUE	147.11334	20	1	Exclude	1.71	13	Medium (~4 m/z)	
TRUE	148.06178	20	1	Exclude	1.617	13	Medium (~4 m/z)	
TRUE	148.09698	20	1	Exclude	0.97	13	Medium (~4 m/z)	
TRUE	150.05872	20	1	Exclude	1.599	13	Medium (~4 m/z)	
TRUE	152.05833	20	1	Exclude	1.611	13	Medium (~4 m/z)	
TRUE	156.07776	20	1	Exclude	0.605	13	Medium (~4 m/z)	
TRUE	161.07297	20	1	Exclude	0.73	13	Medium (~4 m/z)	
TRUE	162.07744	20	1	Exclude	0.863	13	Medium (~4 m/z)	
TRUE	162.09616	20	1	Exclude	4.038	13	Medium (~4 m/z)	
TRUE	162.11411	20	1	Exclude	0.646	13	Medium (~4 m/z)	

TRUE	165.0547	20	1	Exclude	1.31	13	Medium (~4 m/z)	
TRUE	166.08864	20	1	Exclude	1.995	13	Medium (~4 m/z)	
TRUE	168.99248	20	1	Exclude	0.951	13	Medium (~4 m/z)	
TRUE	170.08148	20	1	Exclude	1.049	13	Medium (~4 m/z)	
TRUE	175.1194	20	1	Exclude	0.618	13	Medium (~4 m/z)	
TRUE	176.07057	20	1	Exclude	3.607	13	Medium (~4 m/z)	
TRUE	176.07486	20	1	Exclude	2.497	13	Medium (~4 m/z)	
TRUE	184.07326	20	1	Exclude	1.191	13	Medium (~4 m/z)	
TRUE	187.00047	20	1	Exclude	1.722	13	Medium (~4 m/z)	
TRUE	189.12334	20	1	Exclude	0.885	13	Medium (~4 m/z)	
TRUE	190.05324	20	1	Exclude	2.97	13	Medium (~4 m/z)	
TRUE	192.04839	20	1	Exclude	3.113	13	Medium (~4 m/z)	
TRUE	199.01791	20	1	Exclude	0.742	13	Medium (~4 m/z)	
TRUE	199.02307	20	1	Exclude	1.156	13	Medium (~4 m/z)	
TRUE	205.0997	20	1	Exclude	2.447	13	Medium (~4 m/z)	
TRUE	209.09251	20	1	Exclude	1.804	13	Medium (~4 m/z)	
TRUE	209.09503	20	1	Exclude	1.805	13	Medium (~4 m/z)	
TRUE	215.01663	20	1	Exclude	0.674	13	Medium (~4 m/z)	
TRUE	219.02655	20	1	Exclude	0.663	13	Medium (~4 m/z)	
TRUE	220.07642	20	1	Exclude	1.459	13	Medium (~4 m/z)	
TRUE	220.08636	20	1	Exclude	1.632	13	Medium (~4 m/z)	
TRUE	244.09482	20	1	Exclude	0.681	13	Medium (~4 m/z)	
TRUE	245.07892	20	1	Exclude	5.342	13	Medium (~4 m/z)	
TRUE	256.082	20	1	Exclude	0.766	13	Medium (~4 m/z)	
TRUE	258.1102	20	1	Exclude	0.962	13	Medium (~4 m/z)	
TRUE	258.11264	20	1	Exclude	0.68	13	Medium (~4 m/z)	
TRUE	259.13254	20	1	Exclude	5.974	13	Medium (~4 m/z)	
TRUE	261.03717	20	1	Exclude	0.677	13	Medium (~4 m/z)	
TRUE	263.00851	20	1	Exclude	0.986	13	Medium (~4 m/z)	
TRUE	265.11243	20	1	Exclude	0.619	13	Medium (~4 m/z)	
TRUE	268.10373	20	1	Exclude	1.61	13	Medium (~4 m/z)	
TRUE	268.10782	20	1	Exclude	1.031	13	Medium (~4 m/z)	
TRUE	276.14435	20	1	Exclude	1.231	13	Medium (~4 m/z)	
TRUE	277.13943	20	1	Exclude	0.675	13	Medium (~4 m/z)	
TRUE	277.14313	20	1	Exclude	0.701	13	Medium (~4 m/z)	
TRUE	294.15512	20	1	Exclude	1.233	13	Medium (~4 m/z)	
TRUE	298.09848	20	1	Exclude	0.651	13	Medium (~4 m/z)	
TRUE	298.11035	20	1	Exclude	2.685	13	Medium (~4 m/z)	
TRUE	302.30386	20	1	Exclude	6.117	13	Medium (~4 m/z)	
TRUE	302.31317	20	1	Exclude	6.078	13	Medium (~4 m/z)	
TRUE	305.09637	20	1	Exclude	3.114	13	Medium (~4 m/z)	
TRUE	308.09338	20	1	Exclude	1.585	13	Medium (~4 m/z)	
TRUE	308.10034	20	1	Exclude	0.975	13	Medium (~4 m/z)	
TRUE	312.11166	20	1	Exclude	1.21	13	Medium (~4 m/z)	
TRUE	318.29724	20	1	Exclude	5.97	13	Medium (~4 m/z)	
TRUE	318.30042	20	1	Exclude	6.015	13	Medium (~4 m/z)	
TRUE	325.10614	20	1	Exclude	5.39	13	Medium (~4 m/z)	
TRUE	325.11301	20	1	Exclude	0.708	13	Medium (~4 m/z)	
TRUE	327.09659	20	1	Exclude	5.793	13	Medium (~4 m/z)	
TRUE	328.32593	20	1	Exclude	6.323	13	Medium (~4 m/z)	
TRUE	335.2905	20	1	Exclude	7.214	13	Medium (~4 m/z)	
TRUE	343.1235	20	1	Exclude	0.575	13	Medium (~4 m/z)	
TRUE	346.3313	20	1	Exclude	6.235	13	Medium (~4 m/z)	
TRUE	353.16058	20	1	Exclude	5.432	13	Medium (~4 m/z)	

TRUE	376.31891	20	1	Exclude	6.822	13	Medium (~4 m/z)	
TRUE	381.35123	20	1	Exclude	8.954	13	Medium (~4 m/z)	
TRUE	422.17526	20	1	Exclude	5.074	13	Medium (~4 m/z)	
TRUE	423.0896	20	1	Exclude	0.575	13	Medium (~4 m/z)	
TRUE	435.33362	20	1	Exclude	5.692	13	Medium (~4 m/z)	
TRUE	437.19403	20	1	Exclude	5.917	13	Medium (~4 m/z)	
TRUE	446.15793	20	1	Exclude	0.684	13	Medium (~4 m/z)	
TRUE	507.41199	20	1	Exclude	8.224	13	Medium (~4 m/z)	
TRUE	810.59	20	1	Exclude	6.787	13	Medium (~4 m/z)	
